# Improving the Traits of *Perilla frutescens* (L.) Britt Using Gene Editing Technology

**DOI:** 10.3390/plants13111466

**Published:** 2024-05-25

**Authors:** Sivabalan Karthik, Jia Chae, Seong Ju Han, Jee Hye Kim, Hye Jeong Kim, Young-Soo Chung, Hyun Uk Kim, Jae Bok Heo

**Affiliations:** 1 Department of Molecular Genetic Engineering, Dong-A University, Busan 49315, Republic of Korea; karthik.biotech12@gmail.com (S.K.); cowldk14@gmail.com (J.C.); hsj9587@gmail.com (S.J.H.); kjh48524852@gmail.com (J.H.K.); hjkim83@dau.ac.kr (H.J.K.); chungys@dau.ac.kr (Y.-S.C.); 2 Department of Bioindustry and Bioresource Engineering, Sejong University, Seoul 05006, Republic of Korea

**Keywords:** CRISPR, gene editing, improving traits, perilla species, productivity

## Abstract

Plant breeding has evolved significantly over time with the development of transformation and genome editing techniques. These new strategies help to improve desirable traits in plants. Perilla is a native oil crop grown in Korea. The leaves contain many secondary metabolites related to whitening, aging, antioxidants, and immunity, including rosmarinic acid, vitamin E, luteolin, anthocyanins, and beta-carotene. They are used as healthy and functional food ingredients. It is an industrially valuable cosmetics crop. In addition, perilla seeds are rich in polyunsaturated fatty acids, such as α-linolenic acid and linoleic acid. They are known to be effective in improving neutral lipids in the blood, improving blood circulation, and preventing dementia and cardiovascular diseases, making them excellent crops whose value can be increased through improved traits. This research will also benefit perilla seeds, which can increase their stock through various methods, such as the increased production of functional substances and improved productivity. Recently, significant attention has been paid to trait improvement research involving gene-editing technology. Among these strategies, CRISPR/Cas9 is highly adaptable, enabling accurate and efficient genome editing, targeted mutagenesis, gene knockouts, and the regulation of gene transcription. CRISPR/Cas9-based genome editing has enormous potential for improving perilla; however, the regulation of genome editing is still at an early stage. Therefore, this review summarizes the enhancement of perilla traits using genome editing technology and outlines future directions.

## 1. Introduction

Improving advanced technologies for trait development is crucial to ensuring global food security. Accelerating genomic research and efficiently growing crops are essential for tackling future challenges. New plant breeding technologies are required to develop nutritious foods and climate-resilient crops [1]. These methods include genomic selection, genome editing, and the control of genetic recombination. Genome editing allows for the quick characterization and use of essential genes and alleles for crop improvement [2,3]. This review aimed to improve perilla characteristics by applying gene editing technology. *Perilla frutescens* (L.) Britt (2n = 40) is an annual herbaceous plant belonging to the Lamiaceae family [4,5] and a self-fertilizing crop widely cultivated in South Korea, Japan, China, Vietnam, and India [5]. Perilla, a popular East Asian crop, has two varieties: *P. frutescens* var. *frutescens* is used as an oil crop, whereas *P. frutescens* var. *crispa* is used in Chinese medicine and as a vegetable crop [6]. The seed perilla is specially grown for oil production, whereas the vegetable perilla is cherished for its use in traditional Chinese medicine and as a holistic leafy crop [7]. *Perilla frutescens* var. *frutescens* is a valuable ingredient in Korean cuisine and both its leaves and oils are used [7]. The fresh leaves are often used for wrapping meat and rice, and the pickled form is a popular option [7]. Perilla originally comes from Southern China and is highly valued for its pleasant aroma and medicinal properties [7]. The perilla leaves contain several functional compounds, such as caffeic acid, rosmarinic acid, γ-aminobutyric acid, and luteolin [8]. Perilla seeds contain three types of unsaturated fatty acids including α-linolenic acid (ALA at 54–64%, 18:3), linoleic acid (LA at 11–16%, 18:2), and oleic acid (OA at 14–23%, 18:1) and they also contain 6.7–7.6% of saturated fatty acids, including palmitic acid (16:0) and stearic acid (18:0) [9]. It is a remarkable plant with diverse industrial uses, abundant oil, and high ALA content, making it an important oilseed crop [8]. Perilla seed oil has a higher omega-3 (ALA) content, ranging from 54 to 64%, than other plant oils [10]. In addition, it contains approximately 14% omega-6 (linoleic acid) and omega-9 (oleic acid) fatty acids [10]. These essential fatty acids have been shown to have numerous health benefits, including the prevention of cardiovascular disorders, cancer, inflammation, rheumatoid arthritis, mental illness, diabetes, and insulin resistance. Essential oils (EOs) are aromatic, aliphatic, and terpenoid compounds produced through genetic regulation from mevalonic acid as IPP derivatives or shikimic acid as phenylpropanoids [11,12]. Each variety of essential oil is identified by its unique chemical composition called chemotypes [11,12]. Furthermore, perilla contains different chemotypes based on the synthesis pathways such as the PK-type containing 3-(4-methyl-1-oxopentyl) furan (perillaketone, 5), PA-type containing 1,8-p-menthadiene-7-al (perillaldehyde, 1), EK-type containing 2-(3-methyl-1-oxobutyl)-3-methylfuran (elsholtziaketone, 2), C-type containing 3,7-dimethyl-octanal-2,6-diene (citral, 8), PP-type containing phenylpropanoids, such as 4-methoxy-6-(2-propenyl)-1,3-dioxaindan (myristicin, 9) and 1,2,3-trimethoxy-5-(2-propyl)benzene (elemicin), PL-type containing 3-(4-methyl-3-pentenyl) furan (perillene, 7), and PT-type containing 3-oxo-1,4(8)-*p*-menthadiene (piperitenone, 12) [12,13]. These essential oils have numerous bioactivities, including antibacterial, antiviral, antifungal, anti-inflammatory, antimutagenic, anticarcinogenic, antidiabetic, antiprotozoal, and antioxidant properties [13]. Perilla is used not only as a food ingredient but also as a supplement in animal feed [14]. Its fatty acids have numerous applications in the health and oil industries, as well as in animal husbandry [15]. Therefore, a thorough understanding of fatty acid biosynthesis is essential for the proper utilization of perilla in biomedicine, bioengineering, and animal industries [15]. Hence, including perilla seeds and oil in the diet could have a positive impact on well-being.

Advances in genome editing have accelerated the study of crucial plant traits. However, the perilla improvement requires further optimization. Although CRISPR/Cas-based techniques are primarily used to validate gene function, as technological advances and regulatory frameworks develop to support the commercialization of gene-edited products in some regions, there will be more opportunities for trait improvement [16]. Consequently, CRISPR-based tools have the potential to increase a plant’s resistance against pathogens [17]. Over the last decade, there has been significant progress in CRISPR/Cas-based genome editing in plants, making it ideal to reflect on the lessons learned and explore the latest developments in efficient crop genome editing [18]. The outputs of this pipeline can be integrated into the traditional breeding process to further refine and improve perillas for various traits. Therefore, this review focuses on promising advances in genome editing for perilla trait enhancement.

## 2. Taxonomy of Perilla

The taxonomy of perilla is controversial, and there are varying systems [19,20]. In China, cultivated perilla is classified into five distinct varieties based on its decoration pattern and pollen grain size. These are var. *frutescens*, var. *arguta*, var. *crispa*, var. *auriculato-dentata*, and var. *acuta*. There are different varieties of this plant, but var. *frutescens* and var. *acuta* are commonly used for making fresh vegetables and pickles [20]. On the other hand, var. *crispa* is known for its medicinal properties. Lastly, the seeds of var. *arguta* are used for extracting oil because of their high yield [21]. *Perilla* L. genus has only one species with two varities. *P. frutescens* (L.) Britton var. *frutescens* is grown for oil seed production, and *P. frutescens* (L.) Britton var. *crispa* (Thunb.) W. Deane is used as a spicy vegetable and medicine. Both varieties can cross-fertilize and have green and purple shoots [22].

## 3. Species Classification

**Figure 1:**
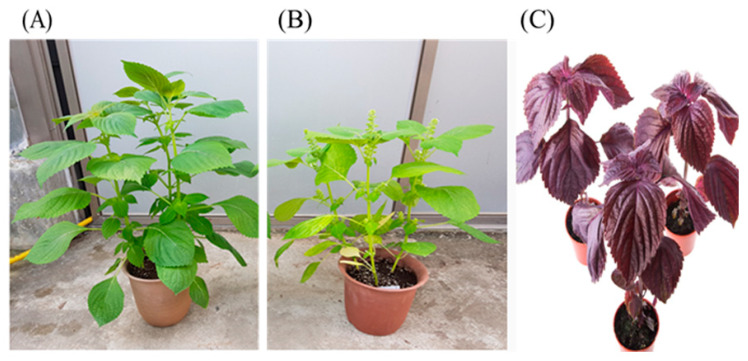
Three kinds of perilla species. (**A**) Leafy perilla. Leafy perilla has a large leaf surface area, displays a late-season phenotype with a late flowering time and has vigorous vegetative growth. (**B**) Seed perilla. Seed perilla has a narrow leaf surface area, blooms more than a month earlier than leafy perilla, and quickly transitions to reproductive growth. (**C**) Shiso. Unlike common perilla varieties, it has a red color.

Perilla species are extensively cultivated in East Asia, particularly in Korea and China. They are classified into three types: leafy, seed, and shiso species (Figure 1), as explained below.

### 3.1. Leafy Perilla

The Namcheon and Manbaek cultivars are commonly grown in the Korean peninsula and are significant herbs and vegetables in Korean cuisine (Figure 1A). Additionally, they are used in Chinese medicine [23]. Certain cultivars are ideal for vegetable preparation due to their increased leaf yield and cyanidin content [11]. These leaves are used in various forms, including fresh, blanched, or pickled in soy sauce or soybean paste, to prepare kimchi and pickles [11]. In Korean-style Western cuisine, these leaves can be used as a substitute for basil [11]. Furthermore, the Bora cultivar has a high level of anthocyanin content and was created by crossbreeding common leaf perilla with perilla seed by Korean breeders.

### 3.2. Seed Perilla

The Dayu cultivar is mainly used as an oil crop for consumption and is commonly cultivated on the Korean peninsula [24]. Deulkkae or Korean perilla seeds are used in two ways: they can be ground into powder or oil(Figure 1B). Roasted deulkkae powder is used as a spice and condiment in soups, seasoned vegetable dishes, noodle dishes, kimchi, and fish cake [24]. It can also be used as a coating or topping for deserts such as yeot and several rice cake varieties. Perilla oil is a popular cooking oil and seasoning made from perilla seeds. The seed powder and oil are also often used in salad dressings and dipping sauces [24].

### 3.3. Shiso

Shiso is also known as *Perilla frutescens* var. *crispa* (Figure 1C). The shiso plant originated from the mountainous regions of Japan [25]. However, the plant has spread worldwide. Different varieties of plant leaves include red, green, bicolor, and ruffled leaves [25]. Shiso comes in several forms, distinguished by the color and shape of the leaves. The red color of shiso is due to the presence of shisonin, an anthocyanin pigment found in the perilla. The first form of shiso studied by Western botanists was the ruffled red shiso, which Carl Peter Thunberg named *P. crispa*, meaning “wavy” or “curly” in Latin. The name *crispa* was later retained when shiso was reclassified as a variety and became widely used in Japanese cuisine [25].

#### Three Varieties of Shiso

There are three varieties of shiso, namely red, green, and bicolor, each used for different purposes.

 *(a)* *Red Shiso*

Red shiso, also known as “akajiso”, is primarily used to give a red color to pickled plums called umeboshi [26,27]. When the leaves of the plant are steeped in “umezu”, the vinegary brine that results from pickling plums, they turn bright red. In the summer, red shiso is used to produce sweet, crimson juice. It may also be used with umezu to make some kinds of sushi. The red shiso plant and its seeds are used in Kyoto to produce “shibazuke”, a fermented eggplant dish [26,27,28]. Red shiso leaves can be dried and crushed to form flakes, which are then mixed with salt to make yukari seasoning. The word “yukari” was initially used by Mishima Foods Co. to refer to their shiso product, and it comes from an old idiom for purple. However, it is now commonly used to denote shiso salt. Red shiso leaf flakes are a popular ingredient for furikake seasoning and are usually sprinkled over rice or mixed with onigiri (rice balls) [26,27,28].

 *(b)* *Green Shiso*

Green shiso, also known as aojiso or ōba (meaning “big leaf”), is a commonly used side dish in Japanese cuisine [28,29]. It is often added to noodle dishes like hiyamugi or sōmen, meat dishes such as sashimi, tataki, and namerō, and tofu dishes like hiyayakko. Moreover, green shiso is a popular garnish for white bait sashimi (shirasu). The leaves of shiso can be used as containers to hold wasabi or tsuma (side dishes) and can be battered on one side and fried to make tempura, which can be served with other fried items. Chopped leaves of shiso are also used as flavor fillings and batters in warm dishes. In Japan, pasta dishes are often topped with dried or freshly chopped shiso leaves combined with raw tarako (pollock roe). Green shiso was initially used as a substitute for basil and has even been used as a topping [28,29].

 *(c)* *Bicolor shiso*

Bicolor shiso, also called Katamen-jiso, is a plant with serrated and pointed leaves in two colors [29]. The top of the leaves was green, while the back side was red. The leaves are flat surfaces that are frequently used to enhance the flavor and color of dishes like soups, side dishes, and first courses [29].

## 4. Improving Perilla Traits

**Figure 2:**
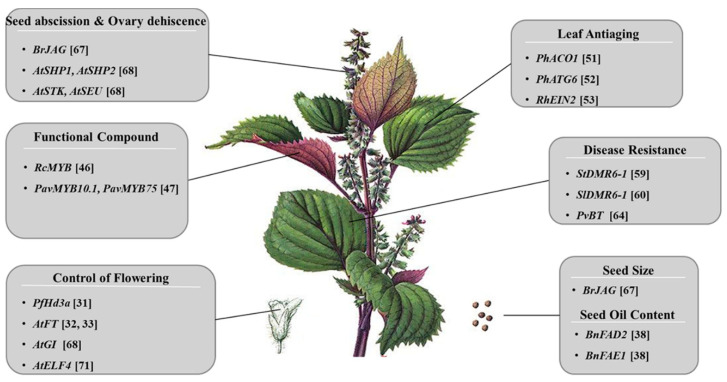
This is a general workflow for genome editing and trait discovery in *Perilla frutescens* using CRISPR/Cas technology.

The following sections explore the prospects of genome editing for improving perilla traits (Figure 2).

### 4.1. Productivity

Flowering management has a significant impact on perilla productivity. Many plant species rely on environmental conditions to control flowering, such as temperature during vernalization and night periods during photoperiodic flowering [30]. Photoperiodic flowering synchronizes flowering time based on day length, which is crucial for adaptation and reproduction [31]. Perilla is a short-day plant and requires specific conditions for flowering [30,31]. It becomes photosensitive at the fourth leaf pair stage, and long nights can encourage flowering [30,31]. Usually, perilla flowering starts after 18–20 days of long nights and continues until it forms seeds after 30 long nights [30,31]. Numerous floral signaling pathways have been identified in Arabidopsis, and distinct flowering regulation gene types respond differently to diverse stimuli and pathways [31]. The convergence of these pathways occurs at the floral integrator genes *FLOWERING LOCUS T* (FT), *SUPPRESSOR OF OVEREXPRESSION OF CONSTANS1* (SOC1), and *TWIN SISTER OF FT* (TSF) [32]. In long-day (LD) and short-day (SD) plants, the essential genes *FT* and *FT* ortholog *Hd3a* integrate various blooming signals [33]. *FT* orthologs have been found in other plants, including peas, kiwifruits, tomatoes, roses, strawberries, and poplar, according to extensive research on a variety of flowering plants [34]. Furthermore, various species have distinct essential night duration requirements for blooming induction, and the movement and speed of the perilla flowering stimulus correspond to photosynthesis, showing phloem transfer [30]. Through orthologous research, Kang et al. (2019) [35] recently identified several genes, including *GIGANTEA* (*GI*), *CONSTANS* (*CO*), and *EARLY FLOWERING 4* (*ELF4*). During the fall and winter, Korean farmers use greenhouses to cultivate perilla. However, short-day conditions during these seasons promote flowering, which can hinder the growth of perilla leaves. To prevent this, farmers usually illuminate their greenhouses, which delays the flowering of perilla plants and allows them to continue harvesting perilla leaves throughout autumn and winter. Although this technique is useful, the installation cost of lighting equipment can be high. Breeding has been suggested as the best approach to solve this problem. Farmers can avoid the cost of installing lighting equipment in their greenhouses by using perilla genetic resources from plant varieties that exhibit delayed flowering. Recently, our research group has been actively working on the mechanism of the flower-related gene *HEADING DATE 3a*, which enhances leaf productivity in perilla, using gene editing technology [31].

### 4.2. Change in Oil Content

In Asia, perilla is a crop used for medicine and oilseed whose seed contains very high quantities of polyunsaturated α-linolenic acid (ALA, omega-3) of up to 60.9%. Omega-3 fatty acids are unsaturated fats with numerous health benefits but are rancid-prone [36]. Despite numerous biotechnological attempts to delay the rancidity of perilla omega-3, none have been successful. A possible solution could be to reduce the C18:3 content and increase the C18:2 and C18:1 content in perilla seeds. Fatty acid desaturases (FADs) accelerate multistep ALA production [36]. A class of enzymes known as fatty acid desaturases (FADs) catalyzes the production of polyunsaturated fatty acids (PUFAs) [36]. Δ7/Δ9 desaturases are the main catalysts for the first-step desaturases in higher plants. Soluble acyl-acyl carrier protein desaturases, or Δ9 desaturases, are the only ones found in all species [36]. There are several reports of ω-3 and Δ12 desaturases in plants. According to Bhunia et al. (2016), these enzymes act as secondary and tertiary desaturases, respectively, accelerating the conversion of oleic acid (C18:1) to linoleic acid (C18:2) and subsequently generating ALA (C18:3) [37]. Long-chain PUFAs are produced by the front-end FADs, which are functionally heterologous enzymes. Plants produce Δ3-desaturated FAs when exposed to FAD4s, a new family of FADs. Most FAD proteins contain three highly conserved histidine motifs that are essential for maintaining their catalytic activity [37]. Recently, it was reported that CRISPR/Cas9 mediated editing of BnFAD2 and BnFAE1 generated novel high-oleic acid germplasms from the CY2 cultivar [38]. Compared to other organs, such as leaf, stem, and root, PfFAD3 showed expression unique to the seed, indicating the preferential accumulation of ALA in the seed [38]. The microsomal oleate 12-desaturase gene (PfFAD3), another alpha-linolenic acid-related gene, was first functionally identified in perilla seeds [36]. Therefore, gene editing on the different FAD genes, namely PfFAD3 and PfFAD2, can increase oil production in perilla.

### 4.3. Increase in Functional Compounds

*P. frutescens* contains hundreds of bioactive functional compounds, two of which are significant phytochemicals: rosmarinic acid (a phenylpropanoid) and perillaldehyde (a monoterpenenoid) [39]. In addition to the antiviral, antibacterial, and anti-inflammatory properties of rosmarinic acid, perillaldehyde has been demonstrated to have anti-inflammatory, antidepressant, antifungal, and antibacterial properties. Enzymes responsible for the biosynthesis of perillaldehyde and rosmarinic acid in *P. frutescens* have been identified. Initially, perillaldehyde is synthesized by the hydroxylation and subsequent oxidation of limonene at the C-7 position [40]. Limonene synthase and a cytochrome monooxygenase catalyze this two-step process of oxidation [40]. On the other hand, rosmarinic acid is proposed to be synthesized from 4-coumaroyl-CoA and 4-hydroxyphenyl acetic acid. The first specific enzyme for rosmarinic acid biosynthesis is rosmarinic acid synthase, which catalyzes the ester formation step [40]. After the formation of 4-coumaroyl-4′-hydroxyphenyl acetic acid, enzymes belonging to the CYP98A family member catalyze the final hydroxylation steps, leading to the production of rosmarinic acid [40]. These enzymes have been cloned and characterized from several plant species, including *Coleus scutellarioides* (Lamiaceae) [40]. However, such enzymes have not been identified in perilla plants. Other functional compounds such as anthocyanins are essential for improving perilla pigmentation. Cyanidins, pelargonidins, delphinidins, petunidins, malvidins, and peonidins are the different categories of anthocyanins. The primary anthocyanins, which range in color from orange and red to purple and blue, are cyanidins, pelargonidins, and delphinidins [41]. Repressing the flavonoid pathway at a single enzyme step results in either a decrease in pigment synthesis or the activation of new compounds in branches upstream of the downregulated gene/enzyme [42]. Gene silencing has more recently been achieved using RNA interference constructs or by the expression of homologous sense RNA or antisense RNA (co-suppression), which is a standard method to downregulate gene expression. Typically, the chalcone synthase (CHS) gene targets the inhibition of the entire flavonoid pathway. Furthermore, antisense CHS constructs were initially used effectively to produce white blooms in tobacco and petunia [43]. When CHS was downregulated, all pigmentation was lost, resulting in white blooms in petunias and chrysanthemums [44]. The overexpression of GMYB10 in transgenic gerbera plants promotes the production of cyanidin, resulting in enhanced pigment accumulation [45]. Transgenic rose lines with white petals showed a significant increase in anthocyanin accumulation upon overexpression of the RcMYB1 transcription factor [46]. MYB transcription factors (TFs) are the most critical transcription level-regulating genes for anthocyanins, which affect phenylpropane metabolism in plants [46]. Repeat sequence variations were divided into four categories: 1R-MYB, R2R3-MYB, 3R-MYB, and 4R-MYB. Certain MYB-TFs (R2R3-MYB) function as activators of anthocyanin biosynthesis, whereas others (R2R3-MYB and R3-MYB) function as repressors [46]. PavMYB10.1 and PavMYB75 upregulate the expression of anthocyanin biosynthesis genes (ABGs), which initiate a cascade of anthocyanin downstream regulators and structural genes in sweet cherries [47]. Hence, the MBW transcription complex containing the MYB, bHLH, and WD40 repeat factors may function as a negative regulator of the anthocyanin signaling pathway. These candidate genes are suitable for gene editing and are well-conserved in many plants, including perilla. 

### 4.4. Leaf Vegetable of Perilla

The length of cut perilla leaves is an essential factor for use as a vegetable. The number of leaves, weight, and other quantitative attributes play significant roles in determining the quality of the leaves. Therefore, in addition to postharvest chemical treatments, molecular and biotechnological approaches must be used to address senescence, organ loss, and other postharvest problems to extend the lifespan of leaves [48]. Perilla vase lifetime can be extended by essential genes that cause senescence and suppress ethylene production, such as *1-aminocyclopropane-1-carboxylic acid synthase* (ACS) and *1-aminocyclopropane-1-carboxylic acid oxidase* (ACO). Transgenic carnations expressing the sense *ACO* gene showed delayed floral senescence linked to decreased ethylene production [49]. In contrast, increased cytokinin levels cause delayed senescence, as demonstrated in transgenic petunia and miniature rose plants overexpressing PSAG12-IPT. This causes the regulation of cytokinin pathways, which in turn leads to delayed senescence and reduced ethylene sensitivity [50]. The ethylene biosynthesis enzyme *1-aminocyclopropane-1-carboxylate oxidase1* (PhACO1) was altered using CRISPR/Cas9 in the petunia variety “Mirage Rose” [51]. The transgenic petunias’ blooms had delayed senescence, which was linked to decreased ethylene production [51]. In contrast, CRISPR/Cas9-mediated gene editing of Petunia’s Autophagy gene 6 (PhATG6) accelerates petal aging by increasing ethylene production and senescence-related gene expression [52]. A new rose knockout mutant for the ethylene-sensitive gene *ETHYLENE INSENSITIVE2* (RhEIN2), important for ethylene signaling, displays ethylene sensitivity and prevents rose blooms from opening [53].

### 4.5. Resistance to Pathogens

Plant pathogens pose a threat to the global food supply, resulting in significant production losses [54]. Climate change is altering pathogen communities, exacerbating this problem [54]. The effective management of plant diseases is crucial for sustainably meeting global food needs. Chemical control is one of the current disease management strategies. Although effective, it may have adverse environmental effects and increase resistance [55]. Conversely, biological management, although more ecologically friendly, often has a low cost-effectiveness and consistency [56]. Nonetheless, the effective management of plant pests and diseases with biological controls and natural resistance has been reported [56]. Consequently, the development of effective disease management strategies requires knowledge of the defense responses and interactions between plants and pathogens [56]. Precise gene alterations without unintended negative consequences are possible with genome editing, particularly using CRISPR-Cas [57]. Many crops are susceptible to various pathogens such as fungi, bacteria, oomycetes, and viruses, which can lead to economic losses. To meet global food demands, it is crucial to develop resistance against these pathogens. Several pathogens, including *Pseudomonas syringae*, *Phytophthora* spp., *Xanthomonas* spp., *Fusarium graminearum*, *Fusarium oxysporum*, *Fusarium solani*, and *Ramularia coleosprii*, significantly affect global perilla production. Downy Mildew Resistance 6 (DMR6) is a potent enzyme that is activated during pathogen infection and belongs to a group of enzymes known as 2-oxoglutarate Fe (II)-dependent oxygenases [58], and it is present in the perilla genome. It can serve as an ideal gene for gene editing to increase the expression of defense genes and elevate SA levels, effectively strengthening the resistance to pathogens in perilla. Silencing of the potato ortholog *StDMR6* enhances resistance to *Phytophthora infestans*, an oomycete pathogen responsible for late blight [59]. The tomato variant *SlDMR6-1* was modified using CRISPR/Cas9 to create mutants that exhibited high resistance to three plant pathogenic bacteria (*Xanthomonas gardneri*, *X. perforans*, and *Pseudomonas syringae* pv. *tomato*) and the oomycete pathogen *Phytophthora capsic* [60]. Mutations in the *dmr6* gene were found to increase the expression of defense genes and elevate SA levels in plants, effectively resisting infections caused by *P. syringae*, *Hyaloperonospora arabidopsidis*, and *Phytophthora capsici* [60]. In tomato, *MAX1* disruption confers resistance against the root-parasitic weed *Phelipanche aegyptiaca* using a CRISPR/Cas9-mediated gene knockout [61]. *LGS1* deletion lines may be more vulnerable to *orobanchol*-sensitive genotypes of *Striga hermonthica* [62]. Furthermore, the *Bacillus thuringiensis* (Bt) method is the most effective technique for insect resistance [63]. The development of transgenic *Populus × euramericana* ‘Neva’ with dual insect resistance is a step forward for poplar advancement [64].

### 4.6. Seed Abscission and Ovary Dehiscence

Due to the aging of farmers, the mechanization of agriculture is urgently needed. Therefore, it is essential to suppress perilla seed shedding to mechanize perilla cultivation. Consequently, seed abscission and ovarian dehiscence are critical factors for trait improvement. Abscission occurs when leaves, fruits, seeds, flowers, petioles, and other organs naturally fall off a plant once it reaches a certain size. Aging and maturity are often accompanied by natural organ abscission [65]. The ability of a mature plant to split along a natural line of weakness and release its contents is called dehiscence. This is typical of sporangia, anthers, and fruits. This occasionally entails the total dissociation of a component. This type of opening is referred to as a dehiscent structure. Decay and predation are two additional mechanisms that allow the release of the structure’s contents; these structures are referred to as indehiscence [66]. Certain flower buds undergo a process known as dehiscence. Certain flower buds (*Platycodon*, *Fuchsia*) undergo a process comparable to dehiscence. However, this process is rarely referred to as dehiscence unless circumscissile dehiscence is included. Anthesis is the term often used to describe the opening of flowers. Abscission may or may not result in a loss of structure during dehiscence. Earlier studies on the use of CRISPR/Cas9-mediated multiplex genome editing to delete all homologous *JAG* led to undifferentiated cell growth in the lateral organs of *Brassica napus* particularly in the pods that surround the ovules [67]. The STK and SHP proteins control the plant’s lignification process and interact with the SEUSS co-repressor [68]. Despite molecular differences in the two developmental pathways, genetic networks that regulate seed abscission and fruit dehiscence are highly conserved [68].

## 5. Progress of Genome Editing Technology in *Perilla frutescens*

Although perilla has immense value as a vegetable or a medicinal material, there has yet to be much research conducted on its trait development through gene editing technology. This is mainly due to the fact that the full genome sequence of perilla has yet to be published. While some research groups have analyzed the genome sequence of perilla, it has yet to be made public. Therefore, to apply a gene editing system in perilla, it is necessary to select useful genes based on comparative genomes through RNA sequencing or use the limited genetic information of perilla previously registered in databases such as NCBI to design guide RNA. If the perilla genome database is made public, many trait development studies could proceed faster through gene editing technology.

In contrast to widely grown crops like rice, soybeans, and tomatoes, perilla research using biotechnology is less prevalent globally. The process of transforming perilla using gene editing technology is also notably challenging due to its low transformation efficiency. Therefore, in order to enhance the characteristics of perillas using gene editing technology, it is essential to first develop technology that can increase the transformation efficiency, which is a unique challenge specific to perillas.

## 6. Conclusions and Challenges for the Future Perspectives

Perilla has a high market potential due to its strong demand in Korea, Japan, and China, and as an oilseed crop with multiple health benefits. The increasing demand for perilla calls requires continued research and the development of new and improved varieties. The use of evolving techniques and technologies is crucial for overcoming obstacles and introducing desirable traits. Although a diverse range of cultivars with advantageous features has been established over time, new tools are required to improve breeding efficiency and overcome challenges such as complicated genetic backgrounds, longer life cycles, and self-incompatibility. Consequently, undesirable side effects in plant breeding can be avoided through genome editing. Knockout and promoter-editing techniques are frequently used in this field. Precise and rapid site-specific genome editing is a promising method for improving traits. The simplicity, productivity, and multiplexing flexibility of genome-editing tools make them highly desirable for specific applications. CRISPR/Cas9-based genome editing tools are considered game-changers in functional genomics and crop breeding for trait enhancement. These powerful technologies have revolutionized our ability to manipulate and comprehend the genetic code, creating unprecedented opportunities for both researchers and farmers. DNA-free editing techniques are essential for developing non-transgenic plants. Although genome editing in perilla is still in its early stages, it has become a popular method for functional genomics and trait improvement research. Genome editing may be particularly beneficial in perilla plants, which face various challenges that limit conventional breeding. However, the complex genetic background of the target and other limitations such as recalcitrance and low efficiency make efficient genome editing challenging. To overcome these barriers, functional genomics and genome engineering research is necessary. 

A deeper understanding of the molecular networks and pathways regulating these traits is required to achieve the future goals of improving perilla traits through genome editing. CAS codon optimization can be used to identify specific and efficient promoters and minimize off-target modifications. The development of genotype-independent regeneration protocols, efficient genotyping, and screening methods for the stable inheritance of target-engineered genes is also crucial. Implementing these cutting-edge tools could revolutionize the improvement of perilla traits. CRISPR can revolutionize agriculture and improve food security in perilla, despite challenges related to reproductive cycles, off-target effects, and regulations. Scientific experts addressing these issues are paving the way for a more sustainable and resilient future for agriculture. Finally, by balancing trade-off side effects with trait enhancement, new perilla varieties can be developed to meet unique breeding demands.

## Data Availability

This review contains referenced information.
